# Eculizumab application during pregnancy in a patient with paroxysmal nocturnal hemoglobinuria: A case report with review of the literature

**DOI:** 10.1002/ccr3.1634

**Published:** 2018-06-27

**Authors:** Lisa Sophie Lauritsch‐Hernandez, Franziska Kraehenmann, Stefan Balabanov, Nina Kimmich

**Affiliations:** ^1^ Department of Obstetrics University Hospital of Zurich Zurich Switzerland; ^2^ Department of Hematology University Hospital of Zurich Zurich Switzerland

**Keywords:** eculizumab, management, maternal and fetal outcome, paroxysmal nocturnal hemoglobinuria, pregnancy

## Abstract

Eculizumab is highly effective in inhibiting complement activation and has successfully shown to prevent complications and to improve quality of life in patients with paroxysmal nocturnal hemoglobinuria (PNH). Its application during pregnancy showed favorable fetal and maternal outcome in the presented case and has proven to be effective without raising safety concerns.

## 1 BACKGROUND

Paroxysmal nocturnal hemoglobinuria (PNH) is a rare disorder of hematopoietic stem cells causing intravascular hemolysis. We report about a patient with PNH treated with eculizumab during pregnancy. Experience on safety and efficacy of eculizumab in pregnancy is scarce. Our case supports its benefit on fetal and maternal outcome so far.

Paroxysmal nocturnal hemoglobinuria is very rare, with a prevalence of 1‐2 cases per million people with a slight female predominance.^1^ PNH can affect women of childbearing age and may be diagnosed during pregnancy for the first time.^2^ It is a non‐neoplastic human disease caused by a somatic mutation of the x‐linked phosphatidylinositol glycan‐complementation class A (PIG‐A) gene in hematopoietic stem cells, which renders red blood cells susceptible to complement‐mediated hemolysis.^3^ It causes a chronic progressive disease characterized by hemolytic anemia, venous thromboembolism, and bone marrow failure.^2^ During pregnancy, terminal complement formation is increased, causing a higher morbidity than in nonpregnant women with PNH, with the requirement for more frequent transfusions.^4^

Paroxysmal nocturnal hemoglobinuria is diagnosed by detection of GPI (Glycosylphosphatidylinositol) negative cells in peripheral blood using flow cytometry with fluorescent aerolysin (FLAER).^5^ In addition, bone marrow analysis (including cytogenetics) is needed to detect other underlying bone marrow disorders (eg, myelodysplastic syndrome or aplastic anemia). Clinical diagnosis can be difficult as PNH has a wide range of symptoms and signs, which can be confounded with various complications seen in pregnancy, for example, preeclampsia, HELLP syndrome, or pregnancy‐associated thrombocytopenia. PNH should always be suspected in pregnant women with unexplained severe anemia, thrombocytopenia, and signs of hemolysis. It is important to distinguish a HELLP syndrome from a PNH crisis, which both can show similar symptoms such as nausea, abdominal pain, and pathological laboratory findings of hemolysis (elevated lactate dehydrogenase (LDH) and indirect bilirubin, low haptoglobin, and low platelets).

Clinical trials in nonpregnant patients with PNH have shown that eculizumab prevents disease complications and improves quality of life and overall survival.^6^, ^7^ Eculizumab is a humanized monoclonal antibody that blocks the activation of terminal complement at C5 and prevents the formation of C5a and the terminal complement complex C5b‐9. The drug has been approved by the FDA in the United States in March 2007 and by the European Medicines Agency in June 2007.

Prior to the approval of eculizumab by the FDA and the European Commission, there were no approved therapeutic options for patients with PNH. Treatment was generally supportive in nature.^8^, ^9^ Without treatment, median survival after diagnosis was limited to 10‐32 years, primarily due to thromboembolic events. Regarding the application of eculizumab during pregnancy, it has shown a low rate of maternal complications up to now. However, there is little expertise in managing pregnant patients with PNH with eculizumab, especially as prospective trials are unlikely to be initiated, as PNH is a very rare disease.^10^, ^11^ The drug is still listed in pregnancy as a category C drug, but potential benefits may outweigh potential risks. It is reported in the literature that eculizumab infusion protocols had to be adjusted in the courses of pregnancies by higher doses or shortening of application intervals. This could possibly be explained by dilutional effects, as plasma volume and complement levels increase during pregnancy.

As there are still few studies about the outcomes of pregnancies in patients with PNH, who were treated with eculizumab, we present such a case and review the existing literature to share clinical information and compare our treatment protocol.

## 2 CASE PRESENTATION

At the age of 25 years, the patient presented herself in her first pregnancy at 37 + 3 weeks of gestation at a small primary care hospital with fever 3 days after rupture of the amniotic membranes. As an incidental finding the patient showed a thrombocytopenia (40 G/L) of unknown origin. Therefore, she was transferred to a secondary care hospital, where a cesarean delivery in general anesthesia was performed the same day, due to a pathological cardiotocography, suspected amniotic infection and thrombocytopenia. Because of postpartum anemia the patient required two red blood cell transfusions. The newborn suffered from asphyxia, followed by infection, and generalized convulsions caused by a cerebral infarction in the right frontotemporal lobe. However, recovery of the neonate was good with normal further development. The mother suffered from a persistent bicytopenia and fatigue after birth. She was followed up in an outpatient setting at a secondary care center, where further diagnostics were initiated. Initial bone marrow biopsy in our patient revealed an increased and megaloblastic erythropoiesis with moderate dyserythropoietic and dysmegacaryopoietic changes. The karyotype of bone marrow cells was normal (46, XX). She was referred to the hematological department of our tertiary care center. A second bone marrow biopsy confirmed the morphological and cytogenetic findings of the first puncture. Furthermore, FLAER of peripheral blood detected a significant PNH population with 94% of granulocytes. Besides, the patient showed gross hematuria. All together, the results were interpreted as classical PNH. At the end, the diagnosis of PNH was stated at an age of 27, not earlier than 18 months after the appearance of her first signs and symptoms during her first pregnancy.

An initial immunosuppression with thymoglobulin for 6 days and cyclosporine for about 2.5 months was ineffective in reducing hemolytic activity, but in contrast caused serum sickness. Therefore, treatment with the relatively new drug eculizumab was initiated 5 months after PNH was diagnosed, and ever since continued biweekly. The PNH clone was measured yearly and decreased from 94% to 80% in the following 2 years. Eculizumab treatment significantly reduced hemolytic activity. Conditions deemed optimal for conception as the patient was planning another pregnancy. For this, prenatal counseling was done by both a specialist in high‐risk pregnancies and a specialized hematologist.

**Figure 1:**
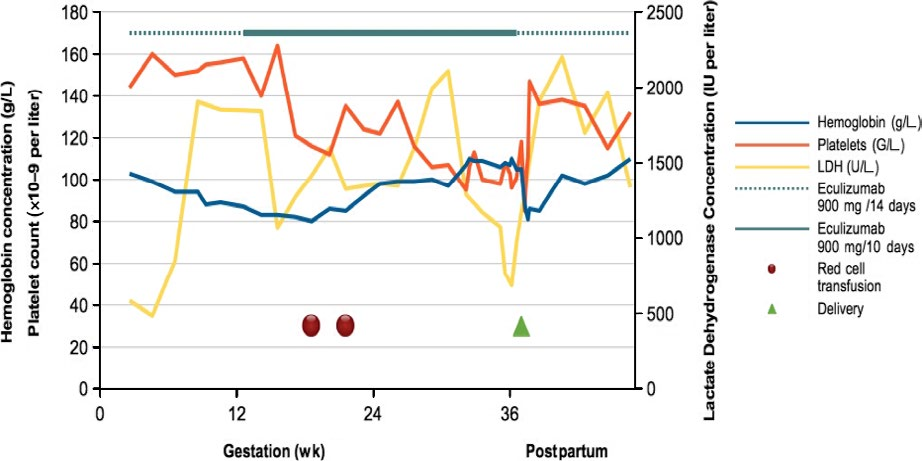
Course of changes in hemoglobin, platelets and lactate dehydrogenase (LDH) concentration during pregnancy

As prophylaxis against venous thromboembolism the patient received an oral anticoagulation with a vitamin K antagonist (Phenprocoumon, 3 mg/d), which was switched to a therapy with low molecular weight heparin (Dalteparin) 6 months later as the patient presented with a positive pregnancy test. Prior to conception, the patient had already received eculizumab continuously for 32 months. Eculizumab was continued throughout the entire pregnancy. The patient had biweekly surveillance at our hematological department before and after pregnancy with application of 900 mg eculizumab and laboratory check‐ups to detect hemolysis. During pregnancy monitoring was intensified to intervals of 10 days as hemolysis occurred in the second trimester, requiring 2 red blood cell transfusions and the application of eculizumab in shorter intervals (10‐11 days) (Table 1). The PNH clone has not been monitored during pregnancy. As a result of the intenser therapy, anemia improved and the level of LDH decreased. Pregnancy monitoring was primarily performed by an obstetrician in private practice. These controls were done in standard intervals of 3‐4 weeks. Ultrasound check‐ups including nuchal translucency, detailed sonomorphology, and Doppler sonography of the great vessels were always inconspicuous. The patient developed gestational diabetes requiring insulin and a mild hypertension in the third trimester, but with no need for antihypertensive therapy, as the blood pressure was only intermittent and marginally high. The patient was referred to our obstetrical department at 36 + 0 gestational weeks because of a rising blood pressure of 150/95 mm Hg. There were no clinical signs of preeclampsia, but altered laboratory results with high LDH, low platelets, and low hemoglobin. As PNH could have been the cause of these alterations but also could have masked a beginning preeclampsia/HELLP syndrome, further clinical monitoring has been done. During clinical observation blood pressure returned to normal and laboratory parameters remained stable, so that a preeclampsia/HELLP syndrome could be excluded. There were also no signs of fetal anemia. At 36 + 0 gestational weeks anti‐C and anti‐M antibodies were detected, which had been negative in the first trimester prior to blood transfusions. In the follow‐up control at 36 + 6 weeks levels of anti‐C were always low (titer 1:16, in tubule 1:4) and anti‐M antibodies no more detectable (with cutoffs of 1:16). Anti‐C antibodies remained positive ever since. Neither thromboembolic events occurred during gestation nor in the postpartum period. A healthy neonate of low birth weight (2730 g) was born by scheduled cesarean at 37 + 0 gestational weeks because of persistent transverse presentation of the fetus. The cesarean was performed in general anesthesia because of thrombocytopenia (106 G/L) combined with significant local hematomas in the patient after daily injection of dalteparin. Cesarean section was uneventful with an estimated blood loss of 400 mL and patient’s recovery was excellent. Hemoglobin level before surgery was 106 g/L and 88 g/L 24 hours after surgery. The course of pregnancy with its therapy and changes in laboratory findings is shown in Figure 1.

**Table 1:** Detailed course of laboratory changes and treatment during pregnancy

Gestation (wk + d)/postpartum	Hemoglobin (g/L)	Platelets (G/L)	LDH (U/L)	Eculizumab (+ interval in days)	Red cell transfusion
2 + 4	103	144	585	900 mg/iv (+14)	
4 + 4	99	160	484	900 mg/iv (+14)	
6 + 4	94	150	850	900 mg/iv (+14)	
8 + 4	94	152	1904	900 mg/iv (+14)	
9 + 2	88	155		900 mg/iv (+12)	
10 + 4	89	156	1851	900 mg/iv (+12)	
12 + 4	87	158	1847	900 mg/iv (+14)	
14 + 1	83	140	1841	900 mg/iv (+10)	
15 + 4	83	164	1068	900 mg/iv (+10)	
17 + 1	82	121	1272	900 mg/iv (+10)	
18 + 4	80	116	1416	900 mg/iv (+10)	1 EC
20 + 1	86	112	1599	900 mg/iv (+10)	
21 + 4	85	135	1326	900 mg/iv (+10)	1 EC
23 + 1	92	124	1346	900 mg/iv (+10)	
24 + 4	98	122	1362	900 mg/iv (+10)	
26 + 1	99	137	1348	900 mg/iv (+10)	
27 + 4	99	116	1595	900 mg/iv (+10)	
29 + 1	100	106	1994	900 mg/iv (+10)	
30 + 4	97	107	2110	900 mg/iv (+10)	
32 + 1	107	95	1288	900 mg/iv (+10)	
33 + 4	109	100	1170	900 mg/iv (+10)	
35 + 1	106	98	1073	900 mg/iv (+10)	
35 + 4	108	108	765	900 mg/iv (+10)	
36 + 0	107	103		900 mg/iv (+10)	
37 + 0 (date of delivery)	105	118			
Postpartum day 5	86	147		900 mg/iv (+14)	
Postpartum day + 11	85	136	1909	900 mg/iv (+14)	
Postpartum day + 25	102	138	2203	900 mg/iv (+14)	
Postpartum day + 39	98	135	1700	900 mg/iv (+14)	
Postpartum day + 53	102	115	1965	900 mg/iv (+14)	
Postpartum day + 67	110	132	1336	900 mg/iv (+14)	

LDH, lactate dehydrogenase.

Direct antiglobulin testing (DAT) turned out positive (++) in the neonate, but no hemolytic reaction requiring therapy occurred in the newborn. As the positive DAT has been interpreted as an A0 incompatibility by the neonatologists, no follow‐up was required. The patient declined breastfeeding, so lactation was inhibited directly postpartum by administration of cabergoline. At the time of dismissal, the patient decided to use condoms for further birth control.

Three weeks after birth anticoagulation was changed back from low molecular weight heparin (dalteparin) to a vitamin K antagonist (phenprocoumon 3 mg/d). During hospitalization, the patient had been co‐managed with our colleges of the hematological department and was followed up there 10 days after dismissal with blood testing and eculizumab administration. Eculizumab has been continued biweekly ever since postpartum.

## 3 DISCUSSION

Ten years ago, PNH was associated with a high mortality and morbidity. Especially in pregnancy mortality rate has shown to be high (8%‐20%), mostly because of fatal thrombotic events. As a consequence, pregnancy was generally discouraged, as maternal mortality has been reported between 8%‐20.8%, primarily due to thromboembolic events, which mainly occurred in the postpartum period. Fetal morbidity and mortality were also high (4%‐9%), which was mainly related to a high rate of preterm birth**.** Birth weight <3 kg has been described in 53% of the cases.^12^, ^13^

When eculizumab was established in 2007, patients suffering from PNH suddenly could regain normal life expectation and quality, so that reproduction became an option for some female patients again. As eculizumab trials have always excluded pregnant patients, the effect of the drug for mother and child was unknown in the beginning. Single case reports on pregnant women suffering from PNH, whom eculizumab had been administered, have suggested a favorable outcome.^14^, ^15^, ^16^, ^17^, ^18^ In our case, we observed comparable positive findings. Results were encouraging but clinical data were not yet sufficient to draw conclusions.

Finally, in 2015, a retrospective study was published evaluating the use of eculizumab in pregnant women and their born children by data of a designed questionnaire, that was sent to members of the International PNH group and physicians participating in the International PNH registry.^11^ Seventy‐five pregnancies in 61 women between 2008 and 2014 were analyzed. No maternal deaths occurred, fetal mortality rate was 4% and premature birth was reported in 29%. The dose of eculizumab or the frequency of its application had to be increased in 54% after the first trimester because of breakthrough hemolysis. The demand for blood transfusion increased from 0.14 monthly units before pregnancy to 0.92 during gestation and platelet transfusions were required in 16 cases. Ten clinically significant bleeding episodes were documented in the third trimester, postpartum hemorrhage in 8 cases. No thrombotic events occurred during pregnancy compared to 2 out of 75 cases (3%) in the postpartum period. In both cases, eculizumab had been stopped after birth and in one case the thrombotic event occurred under LMWH therapy. Sixty‐nine children have been evaluated by development assessment. One child showed transient asymptomatic neutropenia and another preterm born a slightly delayed development of speech. The presence of eculizumab has been detected in several cord‐blood samples, but there was no evidence of complement blockade in the newborn.^15^ So far, the drug was not detected in breastmilk samples.^11^, ^14^ Overall, there are no reported serious adverse effects in neonates, whose mothers were treated with eculizumab during the entire pregnancy, but still more long‐term follow‐up of the infants is needed.^11^ Comparing our data with this study above and other case reports, we find a comparable course. We can report, that in our case no thrombotic events took place, neither during gestation nor in the postpartum period. However, breakthrough hemolysis occurred in our patient and eculizumab had to be administered in shorter intervals. Blood transfusion requirements were increased in pregnancy, as the patient needed two units in her second trimester compared to a transfusion‐free interval since the implementation of eculizumab therapy before pregnancy. The patient developed symptomatic thrombocytopenia, which did not require platelet transfusion, but therefore cesarean had to be performed in general anesthesia. Another similarity we found was the absence of adverse fetal outcome despite eculizumab exposure. Fortunately, the child in our case could be delivered at term as no further complications occurred. Eculizumab had a protective effect without causing any adverse reaction in our case.

Anyhow, we need more specific guidelines for the management of such high‐risk pregnancies. Comparing reviews and case reports, several authors mention the following helpful considerations for the management of pregnancies in patients with PNH. Because the disorder is associated with high maternal and fetal morbidity, all fertile female patients should be counseled about possible risks during pregnancy. The pregnancy controls should ideally be performed by an obstetrician specialized in high‐risk pregnancies in collaboration with a hematologist.^19^ Folic acid should be supplemented, as constant hemolysis causes an increased demand. PNH is associated with iron deficiency, therefore iron replacement is also recommended.^19^ As transfusion requirements are usually more frequent during pregnancy with PNH, it is important to avoid complement‐rich blood products in order to avoid hemolytic crisis and to use washed red blood cells and platelets instead.

Anticoagulation still remains an essential component of the therapy for pregnant patients with PNH, as the risk for thromboembolic events with fatal outcome is relatively high. Most authors recommend the use of low molecular weight heparin, starting in the first trimester and with maintenance throughout the postpartum period of 4‐6 weeks, as we did in our case.

Furthermore, it has to be questioned if anticoagulation is necessary in a patient on eculizumab therapy, as it effectively inhibits hemolysis and therefore might prevent thromboembolic events sufficiently. As breakthrough hemolysis has still been described in patients under eculizumab with the requirement of adjustments in the dosage or intervals, it might not be safe to abstain from anticoagulation. To avoid hemorrhagic events in the peripartal period an adjustment of anticoagulant therapy has to be considered. Monitoring of thrombotic markers is recommended during the treatment.

So far, experiences with eculizumab have been favorable as thromboembolic events and complications could be reduced. As the drug has a long half‐life (10‐12 days) dosing is challenging in gestation due to the physiological changes in blood and plasma volume and coagulation factors. Therefore, parameters of hemolysis as LDH need to be surveilled closely. Nevertheless, there is no clear statement if the drug can be recommended in all pregnant patients with PNH. In a patient with a very big PNH clone size or with signs of hemolysis the drug mostly outweighs the risks for complications associated with PNH. However, it is unknown, if a pregnant patient with a rather small clone size should be treated with eculizumab during pregnancy, considering the possible risk for adverse effects on the neonate. In most patients, eculizumab has been initialized at a granulocyte clone size of 20% and higher.^17^ Birth should be recommended in a hospital with a specialized hematological support and an obstetrical unit with experience in high‐risk pregnancies. Vaginal birth is preferred whenever possible, as the risk of thromboembolic events is lower after vaginal birth compared to a cesarean. Concerning analgesia, it needs to be considered that regional anesthesia might not be feasible in a state of severe thrombocytopenia or because of the application timing of LMWH therapy. Inducing labor may allow a more controlled situation for delivery. Breastfeeding is interpreted as safe as eculizumab is not excreted into breastmilk.^11^ However, much more case reports will be necessary for a better understanding of the effects of the drug in patients and their offspring to finally be able to establish general guidelines of managing pregnant patients with PNH in the best manner.

## 4 CONCLUSION

Summing up the use of eculizumab during pregnancy in our case and the reported cases so far, it has shown favorable results, which are encouraging for female patients with PNH who wish to become pregnant. Nevertheless, more evidence is required to prove the safety of eculizumab for mother and child and to establish a more effective management during pregnancy.

## CONFLICT OF INTEREST

None declared.

## AUTHOR CONTRIBUTIONS

LL: involved in data analysis and interpretation, drafting of the manuscript, and final approval of the manuscript. NK: performed acquisition of data, drafting and revising the manuscript, and final approval of the manuscript. FK: carried out obstetrical care of the patient and revision of the manuscript. SB: carried out hematological care of the patient and revision of the manuscript. All authors agree to be accountable for all aspects of the work.
